# Simultaneous determination of steroid hormones and pharmaceuticals in killer whale (*Orcinus orca*) faecal samples by liquid chromatography tandem mass spectrometry

**DOI:** 10.1093/conphys/coad081

**Published:** 2023-11-11

**Authors:** Andrew R S Ross, Xiangjun Liao, Tanya M Brown

**Affiliations:** Fisheries and Oceans Canada, 9860 West Saanich Road, Sidney, BC, V8L 4B2, Canada; Fisheries and Oceans Canada, 9860 West Saanich Road, Sidney, BC, V8L 4B2, Canada; Fisheries and Oceans Canada, 9860 West Saanich Road, Sidney, BC, V8L 4B2, Canada

**Keywords:** Killer whale faeces, liquid chromatography–tandem mass spectrometry, steroid hormones, pharmaceuticals and personal care products

## Abstract

We describe a non-invasive method for profiling selected hormones, pharmaceuticals and personal care products (PPCPs) in killer whales (*Orcinus orca*) based on analysis of faecal samples by liquid chromatography tandem mass spectrometry (LC–MS/MS). The method targets 21 compounds of interest including glucocorticoids, mineralocorticoids, androgens, estrogens, progestogens, selective serotonin uptake inhibitors and an antibacterial/antifungal agent. This method is suitable for routine simultaneous determination of target compounds in killer whale faecal samples as well as validation of immunoassays for the detection and measurement of steroid hormones in faeces. The optimized method involves extraction of freeze-dried faecal material with reagent alcohol and water followed by isolation of the analytes using solid phase extraction with hydrophilic–lipophilic balance cartridges and liquid–liquid extraction with methyl tertiary-butyl ether. Reconstituted extracts were analysed by LC–MS/MS using an electrospray ionization interface. Method limit of quantification ranged from 0.06 to 45.2 ng/g in freeze-dried faecal samples. Except for sertraline, triclosan and estradiol (which was not recovered at the lowest spiked concentration), average intra- and inter-day precisions were within 10%, and average recoveries were between 89.3% and 129.3%, for faecal samples spiked with 5.3, 26.7 or 133 ng/g of each analyte. The method was applied successfully to the analysis of hormones and PPCPs in whale faeces during which 17α-hydroxyprogesterone, a common intermediate in steroid biosynthesis that cross-reacts with precursors and sulphated conjugates in immunoassays, was identified and quantified in all samples.

## Teaser

A new method has been developed to analyze hormones, pharmaceuticals and personal care products in killer whale fecal (scat) samples. Using this method, key indicators of health and exposure to harmful chemicals can now be measured quickly and accurately in Southern Resident Killer Whales without disturbing these endangered marine mammals.

## Introduction

Steroid hormones influence animal behaviour and are key indicators of stress in animal behavioural ecology (Dufty *et al.,* 2002; Möstl and Palme, 2002; Godwin *et al.,* 2003). Remote sampling of urine or faecal material avoids disturbing or restricting the behaviour of wild fauna and is commonly used for hormonal studies in free-ranging animals (Schwarzenberger, 2007; Wasser *et al.,* 2010; Ganswindt *et al.,* 2012). Faecal hormone measurements have been used as a non-invasive approach to assess health, evaluate reproduction status and identify physiological responses to disturbance in whales (Ayres *et al.,* 2012; Lundin *et al.,* 2016; Lemos *et al.,* 2022). As apex predators and iconic species, killer whales (*Orcinus orca*) are of great cultural and ecological importance, acting as sentinels of marine ecosystem health. The Southern Resident Killer Whale (SRKW) population, which ranges in the Salish Sea and along the West Coast of the USA and Canada, suffered an unexplained decline of almost 20% in the late 1990s and was subsequently listed as endangered under the Canadian Species at Risk Act in 2001 (Baird, 2001) and the US Endangered Species Act in 2005 (Wiles, 2016). Only 75 individuals (25 in J Pod, 16 in K Pod and 34 in L Pod) remain in the current population as of 30 June 2023. Because skin and blubber biopsy sampling is invasive and no longer permitted, interest in monitoring the health of the remaining SRKW population has led to a demand for analysis of hormones and other bioindicators of health in faecal (or scat) samples collected using non-invasive methods. In this study, we aim to optimize and validate a liquid chromatography tandem mass spectrometry (LC–MS/MS) steroid hormone assay for killer whale faecal samples. The steroid hormones selected in our study include hormones from the four major steroid hormone classes (corticoids, androgens, estrogens and progesterones) that are commonly measured in cetaceans (Ayres *et al.,* 2012, Lemos *et al.,* 2022, Lemos *et al.,* 2020, Lundin *et al.,* 2016; Steinman *et al.,* 2020) and have a common metabolic pathway (Miller, 1988; Galligan *et al.,* 2018; Norris and Carr, 2020). These include hormones that are involved in regulating metabolism and the stress response (glucocorticoids), male sex hormones (androgens), female sex hormones (estrogens) and the luteal phase of the estrous cycle and are precursors to androgens and corticosteroids (progesterones) (Miller, 1988; Norris and Carr, 2020).

Steroid hormones in different cetacean matrices have commonly been measured using radio immunoassays (RIA) and enzyme immunoassays (EIA) that rely on the binding of antibodies to hormones of interest or their metabolites, thus enabling their concentrations to be determined in processed samples. RIA has been used to measure glucocorticoid metabolites in scat samples collected from endangered SRKWs to determine if they have become prey limited due to a reduction in the abundance and availability of Chinook salmon (*Oncorhynchus tshawytscha*), which is one possible explanation for their decline (Ayres *et al.,* 2012). EIA has been used to measure cortisol, corticosterone, 5β-tetrahydrocorticosterone, androstenedione, 5α-tetrahydrocorticosterone, testosterone, 5α-dihydrotestosterone, testosterone-sulphate, testosterone-glucuronide and dehydroepiandrosteronein in faecal samples from killer whales under managed care to obtain insights into the physiologic response of clinically healthy killer whales to stress (Steinman *et al.,* 2020). Enzyme-linked immunosorbent assay (ELISA) has also been used to quantify progestin, androgen, progesterone, testosterone and cortisol in faecal samples collected from gray whales (live and stranded) (Lemos *et al.,* 2020). Although these methods are sensitive and enable high throughput determination of hormones, specificity can be compromised by cross-reactivity with non-target analytes, which may produce misleading results. Furthermore, immunoassays are limited to the analysis of a single analyte per assay, requiring the researcher to perform independent assays for each hormone of interest (Weltring *et al.,* 2012). Unlike immunoassays, LC–MS/MS can provide specific, high-throughput analysis of multiple hormones through the assignment of one or more unique selected reaction monitoring or multiple reaction monitoring (MRM) transitions to each analyte. LC–MS/MS has been applied to the analysis of hormones in faecal samples from a variety of mammals, including endogenous steroid hormones and their metabolites in the faeces of New World primates (Weltring *et al.,* 2012), faecal testosterone and faecal estrogens in wild female and male baboons *(Papio cynocephalus)* (Gesquiere *et al.,* 2014), faecal metabolites of endogenous steroids in Taiwanese pangolin (*Manis pentadactyla pentadactyla*) (Arora *et al.,* 2020) and faecal cortisol metabolites in free-ranging Iberian ibex (*Capra pyrenaica*) (Molina-Garcia *et al.,* 2018). To our knowledge, LC–MS/MS has not yet been applied to the study of hormone profiles in killer whale faecal samples.

**Table 1:** Instrument conditions

LC condition	Mobile phase^a^	A		B				
	Gradient profile	Time (min)	0	4	15	22	27.5	35
		A%	90	90	10	10	90	90
		B%	10	10	90	90	10	10
	Total flow	0.2 ml min^−1^						
	Column	Xterra MS C18 column (100 × 2.1 mm, 3.5 μm)						
MS condition	Ionization mode	ESI						
	Collision gas	8						
	Curtain gas	30						
	Ion source gas 1	30						
	Ion source gas 2	40						
	Ion spray voltage	±4500						
	Temperature (°C)	400						

a A, 0.1% (v/v) formic acid in water; B, 0.1% (v/v) formic acid in methanol.

**Table 2:** Compound number, name, retention time, MS/MS acquisition parameters and corresponding internal standards for analytes

Compound Name	Retention time (min)	Precursor ion (m/z)	Product ions (m/z) (A/B)^a^	DP (volts)	CE (volts)	CXP (volts)	Internal standard
Estriol	20.49	289	107/253	121/136	41/27	20/26	Estriol-d_2_
Aldosterone	20.77	361.1	343.1/315.2	130/100	26/15	26/14	Cortisone-d_8_
Fluoxetine	20.75	310.3	44.1/148	46	13	16	Fluoxetine-d_5_
Sertraline	20.96	306.1	275.1/159	31	17/33	14	Sertraline-d_3_
Cortisone	21.23	361.1	163/121	26	31/41	30/46	Cortisone-d_8_
Cortisol	21.59	363.1	121/91	96	33/91	16/20	Cortisol-d_2_
11-Ketotestosterone	21.76	303.3	121/259.2	70	32	14	11-Ketotestosterone-d_3_
Corticosterone	22.48	347.1	121.1/91	70/26	32/77	10/38	Corticosterone-d_8_
11-Deoxycortisol	22.59	347.2	97.2/109	121	45/39	40/24	11-Deoxycortisol-d_7_
Androstenedione	23.09	287.1	97/109	71	31/33	24/26	Androstenedione-13C_3_
Estrone	23.24	271.3	133/159.3	91/75	31/30	26/10	Estrone-^13^C_3_
17β-E2	23.3	273	107/255	81	43/21	46/36	Estrone-^13^C_3_
11-Deoxycorticosterone	23.39	331.2	97.1/109	46	31/33	12/42	11-Deoxycorticosterone-d_7_
Testosterone	23.61	289.1	109.1/97	46	33/31	24/38	Testosterone-d_3_
17-Hydroxyprogesterone	23.7	331	97/109	116	41/39	22/44	17-Hydroxyprogesterone-d_8_
DHEA	23.93	289.2/271.1	253	136/151	15/19	14/38	DHEA-d_6_
17α, 20β-dihydroxyprogesterone	23.93	333.3	97.1/109	91	35/41	22/24	Progesterone-d_9_
Progesterone	24.94	315.1	109.2/97.2	66	33/39	30/54	Progesterone-d_9_
Androsterone	25.22	291.1/273.3	255/147.1	61/146	21/31	14/30	Androsterone-d_4_
Triclosan	26.51	286.8	35	−150	−54	−18	Triclosan-^13^C_12_
DHEAs	31.43	367.2	97/79.6	−150	−54	−18	Not applicable

CE, collision energy; CXP, collision cell exit potential; DP, declustering potential.

^a^A, quantitative ion; B, qualitative ion.

Pharmaceuticals and personal care products (PPCPs) constitute a diverse array of compounds including antibiotics, antimicrobial agents and synthetic musks. These compounds have raised significant concerns in recent years due to their ongoing use and potential to cause ecological harm in the environment. Many PPCPs remain biologically active after leaving the body, leading to the possibility of adverse effects in non-target organisms including aquatic biota (Ramirez *et al.,* 2009; Liu and Wong, 2013; Mottaleb *et al.,* 2015; Arpin-Pont *et al.,* 2016). Studies have suggested that some PPCPs have the potential to bioaccumulate and cause abnormalities in fish (Jobling *et al.,* 1998; Vajda *et al.,* 2008; Vajda *et al.,* 2011), whereas others have concluded that PPCPs may interfere with the thyroid axis in zebra fish (Pinto *et al.,* 2013), impede swimming behaviour in fathead minnow (Fritsch *et al.,* 2013) or be responsible for the formation of adducts with haemoglobin and/or protein breakdown products (Mottaleb *et al.,* 2006; Mottaleb *et al.,* 2008; Mottaleb *et al.,* 2012; Mottaleb *et al.,* 2015). PPCPs with anti-seizure, anti-depressant and endocrine-disrupting effects are often found in fish liver and muscle tissue. Sertraline has been quantified in both tissues, and fluoxetine in fish liver (Arpin-Pont *et al.,* 2016). Triclosan and its transformation product methyl-triclosan have also been found in fish (Rudel *et al.,* 2013). During daylight hours, SRKWs spend about half their time hunting for fish, with salmonids (mainly Chinook) constituting over 90% of their diet (Ford *et al.,* 2016). The potential for dietary exposure of SRKW to a range of environmental contaminants including PPCPs and polybrominated diphenyl ethers has prompted research into the measurement of these contaminants in whale faeces as part of the Government of Canada Whales Initiative. PPCPs such as triclosan, sertraline and fluoxetine all have been found in fish tissues (Rudel *et al.,* 2013; Arpin-Pont *et al.,* 2016) and are, therefore, principal targets for analysis in SRKW faecal samples.

The aim of this study was to optimize and validate a specific and reliable method for simultaneous detection and quantification of multiple steroid hormones and PPCPs in SRKW faecal samples based on LC–MS/MS. The list of 21 analytes includes glucocorticoids (cortisol, cortisone and 11-deoxycortisol), a mineralocorticoid (aldosterone), androgens (dehydroepiandrosterone, androstenedione, androsterone and testosterone), estrogens (estrone, estriol and 17β-estradiol), progestogens (progesterone and 17α-hydroxyprogesterone) and other steroid hormones (17α,20β-dihydroxyprogesterone, corticosterone, 11-ketotestosterone, 11-deoxycorticosterone and the sodium salt of dehydroepiandrosterone sulphate) as well serotonin reuptake inhibitors (fluoxetine and sertraline) and an antibacterial/antifungal agent (triclosan). Specific objectives were to (i) improve on existing limits of detection by using solvent extraction, solid phase extraction (SPE) and liquid–liquid extraction (LLE) procedures to optimize recoveries and reduce matrix effects, and (ii) validate the new method in terms of sensitivity (method limit of quantification), linearity, precision, recovery and freedom from matrix interferences. The resulting LC–MS/MS method provides a new tool for rapid profiling of key hormones and PPCPs in killer whale faecal samples (including the endangered SRKW) and for validating immunological methods such as RIA and EIA for the analysis of such compounds in whale faeces.

**Figure 1:**
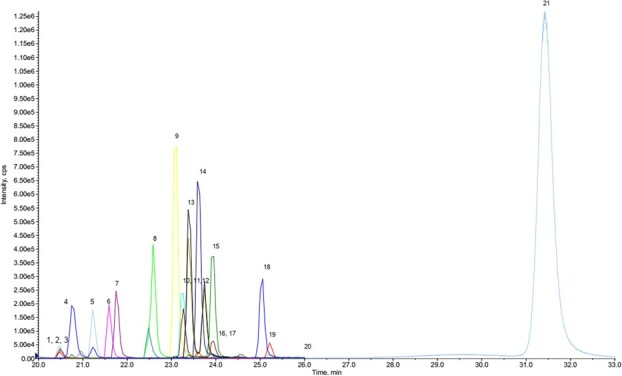
Chromatograms of hormone and PPCPs in standard mix. 1. Estriol; 2. Aldosterone; 3. Fluoxetine; 4. Sertraline; 5. Cortisone; 6. Cortisol; 7. 11-Ketotestosterone; 8. Corticosterone; 9. 11-Deoxycortisol; 10. Androstenedione; 11. Estrone; 12. 17β-E2; 13. 11-Deoxycorticosterone; 14. Testosterone; 15. 17-Hydroxyprogesterone; 16. DHEA; 17. 17α,20β-dihydroxyprogesterone; 18. Progesterone; 19. Androsterone; 20. Triclosan; 21. DHEAs

## Materials and Methods

### Samples

Six faecal samples from killer whales under managed care were provided by SeaWorld (SeaWorld and Busch Gardens Species Preservation Laboratory, San Diego, CA, USA). Faecal samples were collected from animals as part of routine husbandry management. All collection procedures were approved by the SeaWorld Parks and Entertainment Incorporated Research Review Committee and were performed in accordance with the United States Animal Welfare Act for the care of marine mammals. All samples were frozen at −80°C until analysis.

### Chemicals and reagents

Details of the chemicals and reagents used can be found in Supplementary Material. Standards received in solid form were first dissolved in methanol (MeOH) at a concentration of 1 mg/ml, then diluted to 100 μg/ml with MeOH and the resulting stock solutions stored at −20°C. A standard mixture containing each native analyte at a concentration of 100 ng/ml was prepared in 20:80 (v/v) MeOH/water. Seven working calibration standard solutions (0.1, 0.2, 0.5, 1, 2, 5 and 10 ng/ml) were obtained by diluting the appropriate amount of the standard mixture in 20:80 MeOH/water containing each internal standard at 10 ng/ml. Spiking at various concentrations was obtained by combining aliquots of stock solutions with MeOH.

### Extraction of faecal samples

Frozen faecal samples received from Seaworld were lyophilized (Freezone 4.5 plus, Labconco) before extraction, which was carried out according to Wasser et al. (Wasser *et al.,* 2010) and Weltring et al. (Weltring *et al.,* 2012) with minor modifications. Briefly, 5 ml of reagent alcohol was added to the tube containing ~40 mg of freeze-dried sample and an appropriate amount of the labelled internal standard and the mixture vortexed for 5 minutes followed by the addition of another 5 ml of reagent alcohol in the same tube and vortexing for another 5 minutes. Then 3 ml of water was added to the mixture, which was vortexed for 5 minutes and sonicated for 30 minutes. After centrifugation, the supernatant was decanted and this extract stored at −20°C.

**Table 3:** Signal suppression for target compounds using different sample extraction procedures

Compound	Signal suppression (%)		Ratio
	SPE + LLE	SPE only	
Estriol	15	57	3.8
Aldosterone	12	45	3.8
Fluoxetine	−22	−11	0.5
Sertraline	−50	−11	0.2
Cortisone	13	49	3.8
Cortisol	19	47	2.5
11-Ketotestosterone	18	47	2.7
Corticosterone	22	43	1.9
11-Deoxycortisol	23	44	1.9
Androstenedione	44	47	1.1
Estrone	29	49	1.7
17β-E2	35	58	1.6
11-Deoxycorticosterone	26	37	1.4
Testosterone	24	37	1.5
17-Hydroxyprogesterone	37	48	1.3
DHEA	24	47	1.9
17α, 20β-dihydroxyprogesterone	25	36	1.4
Progesterone	22	16	0.7
Androsterone	20	35	1.8
Triclosan	41	82	2
DHEAs	−118	−115	1

SPE: solid phase extraction with water and methanol.

SPE + LLE: solid phase extraction followed by liquid–liquid extraction with MTBE.

Ratio: the ratio of matrix suppression for SPE alone to that for SPE + LLE.

**Table 4:** Method limit of quantification (ng/g) for target compounds using different extraction methods

Compound	Method	
	SPE + LLE	SPE only
Estriol	4.89	11.48
Aldosterone	6.48	11.66
Fluoxetine	0.06	0.35
Sertraline	0.73	4.17
Cortisone	0.87	1.99
Cortisol	2.17	4.58
11-Ketotestosterone	2.19	2.86
Corticosterone	5.06	7.78
11-Deoxycortisol	2.07	1.74
Androstenedione	1.23	2.29
Estrone	2.96	6.05
17β-E2	21.19	42.63
11-Deoxycorticosterone	0.97	1.86
Testosterone	1.79	2.72
17-Hydroxyprogesterone	3.61	7.9
DHEA	42.52	28.63
17α, 20β-dihydroxyprogesterone	2.8	3.5
Progesterone	4.39	6.59
Androsterone	12.32	6.91
Triclosan	3.11	30.94
DHEAs	1.58	4.57
Min	0.06	0.35
Max	42.52	42.63

SPE: solid phase extraction with water and methanol.

SPE + LLE: solid phase extraction followed by liquid–liquid extraction with MTBE.

**Table 5:** Calibration slopes for target compounds in different media after applying isotope correction

Compound	Solvent	SPE + LLE	SPE only	RSD (%)
Estriol	0.132	0.113	0.128	8.06
Aldosterone	0.111	0.101	0.117	7.37
Fluoxetine	0.072	0.081	0.084	7.99
Sertraline	0.756	0.812	0.8	3.74
Cortisone	0.088	0.084	0.086	2.15
Cortisol	0.253	0.261	0.274	4.04
11-Ketotestosterone	0.092	0.09	0.088	2
Corticosterone	0.171	0.169	0.187	5.62
11-Deoxycortisol	0.154	0.149	0.163	4.57
Androstenedione	0.128	0.128	0.132	1.79
Estrone	0.102	0.103	0.107	2.54
17β-E2	0.011	0.009	0.01	9.14
11-Deoxycorticosterone	0.213	0.211	0.217	1.43
Testosterone	0.078	0.077	0.08	2.1
17-Hydroxyprogesterone	0.063	0.06	0.068	6.6
DHEA	0.077	0.062	0.065	11.39
17α, 20β-dihydroxyprogesterone	0.053	0.048	0.041	13.1
Progesterone	0.051	0.05	0.053	2.85
Androsterone	0.092	0.095	0.101	4.76
Triclosan	0.068	0.072	0.082	9.97
DHEAs	6.76E+06	1.08E+07	1.10E+07	25.13

SPE: solid phase extraction; SPE + LLE: solid phase extraction followed by liquid–liquid extraction with MTBE.

**Table 6:** Calibration intercepts for target compounds in different media after applying isotope correction

Compound	Solvent	SPE + LLE	SPE only	RSD (%)
Estriol	0.132	0.113	0.128	8.06
Aldosterone	0.0005	0.0005	−0.0075	−214.71
Fluoxetine	−0.0016	−0.0011	−0.0009	−30.97
Sertraline	−0.0153	−0.0003	−0.0228	−89.4
Cortisone	−0.0002	0.0022	0.0076	123.75
Cortisol	0.0294	0.035	0.0435	19.74
11-Ketotestosterone	0.002	0.0059	0.0135	82.13
Corticosterone	0.0009	0.009	0.0065	75.43
11-Deoxycortisol	0.0008	0.004	0.0035	62.53
Androstenedione	0.0027	0.0168	0.0232	73.86
Estrone	0.0009	0.0037	0.0038	59.59
17β-E2	0.0039	0.0065	0.0057	24.52
11-Deoxycorticosterone	−0.0007	0.0065	0.0095	102.5
Testosterone	0.0007	0.0021	0.0018	48.76
17-Hydroxyprogesterone	0.0006	0.0093	0.0096	78.35
DHEA	0.0035	0.0178	0.007	79.25
17α, 20β-dihydroxyprogesterone	0.0015	0.0004	0.0002	96.13
Progesterone	0.051	0.0498	0.0527	2.85
Androsterone	0.0008	−0.0003	0.0004	175.92
Triclosan	−0.0009	−0.0011	0.0026	1169.01
DHEAs	−6.31E+05	6.21E+05	5.82E+06	176.63

SPE: solid phase extraction; SPE + LLE: solid phase extraction followed by liquid–liquid extraction with MTBE.

**Table 7:** Intra-day and inter-day recovery and precision for spiked freeze-dried faeces of killer whales

	Spiked level 1 (5.3 ng/g)				Spiked level 2 (26.7 ng/g)				Spiked level 3 (133 ng/g)			
Compound	Intra-day		Inter-day		Intra-day		Inter-day		Intra-day		Inter-day	
	RE (%)	RSD (%)	RE (%)	RSD (%)	RE (%)	RSD (%)	RE (%)	RSD (%)	RE (%)	RSD (%)	RE (%)	RSD (%)
Estriol	NA	NA	NA	NA	105.9	18.7	121.1	40.5	75.7	14.9	118	38.7
Aldosterone	116.2	0.6	104.5	7.2	96.1	2.2	92.8	7.2	84.5	1.7	89.6	5.5
Fluoxetine	91.2	3.8	88.1	7.9	97.2	5	90.6	4.4	88.7	0.8	89.3	4.3
Sertraline	48.6	2.9	68.3	30.8	93.5	1.8	93.7	5.8	102.1	11	95	14
Cortisone	120.4	8.6	122.3	4.4	117.1	1.6	121.2	6.7	130	0.7	130.3	4.2
Cortisol	99	3.3	89.3	7.6	117.1	5	111.9	5.8	118.2	1.9	115.6	7.9
11-Ketotestosterone	97.3	4	95	12.1	107	1.7	109.1	5.4	108.8	4.8	106.9	0.8
Corticosterone	104.1	9.6	110.8	5.4	120.9	3	123.9	2.3	121.5	4.8	115.6	5.7
11-Deoxycortisol	113.5	6.7	117.7	5.4	116.1	4	118.6	7.1	111	2.7	112.8	4.1
Androstenedione	101.2	3	88.6	6.3	106.2	6.2	97.6	6	102.8	5.9	97.7	6.7
Estrone	91.5	7.7	113.4	16.7	112.3	1	110.2	3.7	106.7	3	102.8	3.8
17β-E2	101.1	7.8	114.4	13.1	110.4	10.6	113.2	3.5	103.8	4.2	104.6	2
11-Deoxycorticosterone	124.8	3	114.5	8.5	122.2	5.5	125.3	3.6	117.8	3.3	120.1	4.7
Testosterone	102.1	2.4	107.2	2.7	96.9	0.5	100.5	6.9	96.4	1.9	96.1	1.9
17-Hydroxyprogesterone	117.6	1.4	135.8	10.8	117	9	123.7	4.4	119.1	6.9	117	7.2
DHEA	125	10.7	122.4	4.5	111.1	9.8	104.2	4	112.2	5.6	90	20.8
17α, 20β-dihydroxyprogesterone	114.8	6.7	112.2	2.5	133	4.6	123	8.2	140	9.3	122.2	13
Progesterone	121.9	8.9	112.7	8.5	121.6	0.9	121.4	5.3	115.4	3.9	108.4	6.6
Androsterone	110.6	3.7	105.9	3.5	112.3	1.4	104.8	8	112.7	0.7	105.2	6.6
Triclosan	85.9	41.1	157.2	40.4	102.1	5.5	128.9	30.1	91.9	7.4	97.6	6.6

RE, recovery; RSD, relative standard deviation.

**Table 8:** Method limit of detection (ng/g) for analysis of hormones in mammalian faecal samples

Compound	Method		
	This study (killer whale, *O. orca*)	Gesquiere et al. (baboons, *P. cynocephalus*)	Weltring et al. (New World primates, *Cebus capucinus*)
Estriol	1.47		19.2
Aldosterone	1.94		
Fluoxetine	0.02		
Sertraline	0.22		
Cortisone	0.26		91.8
Cortisol	0.65		18
11-Ketotestosterone	0.66		
Corticosterone	1.52		99
11-Deoxycortisol	0.62		
Androstenedione	0.37	0.25	47.4
Estrone	0.89	5	88.2
17β-E2	6.36	0.25	35.4
11-Deoxycorticosterone	0.29		
Testosterone	0.54	0.25	54.6
17-Hydroxyprogesterone	1.08		
DHEA	12.76	2.5	
17α, 20β-dihydroxyprogesterone	0.84		
Progesterone	1.32		50.4
Androsterone	3.7	1.5	210.6
Triclosan	0.93		

Extracts were subjected to SPE using cartridges Oasis HLB (60 mg, 3 ml) alone or in combination with LLE to reduce matrix effects and enhance measurement of analytes. SPE cartridges were conditioned sequentially with 4.7 ml of MeOH and 4.7 ml of water before sample loading. After washing with 4.7 ml of water, steroid hormones and PPCPs were eluted with 4 ml of MeOH and collected in a single fraction. The collected SPE fraction was dried with N_2_ and reconstituted in 500 μl of 20:80 MeOH/water, of which 300 μl was transferred to a vial insert for LC–MS/MS analysis. The remaining 200 μl of reconstituted solution was further isolated by LLE with 800 μl of MTBE. The upper layer from LLE was collected, dried with N_2_, reconstituted with 200 μl of 20:80 MeOH/water and subjected to LC–MS/MS analysis.

### Liquid chromatography tandem mass spectrometry

Isolated sample fractions were analysed by LC–MS/MS using a PerkinElmer Series 200 high-performance liquid chromatography (HPLC) system coupled to an API5000 triple-quadrupole tandem mass spectrometer fitted with a TurboIonSpray™ source (ABSciex, Concord, ON, Canada). Compounds of interest were separated on a reversed phase C18 column (Xterra® MS C18 column, 100 × 2.1 mm, 3.5 μm, Waters, Milford, MA, USA) protected by a VanGuard® cartridge of the same material (Xterra® MS C18; 5 × 2.1 mm, 3.5 μm, Waters, Milford, MA, USA). The column oven was set at 40°C, and the injection volume was 50 μl. Optimized instrumental conditions are shown in Table 1. With the exception of DHEAs and triclosan, which were analysed in negative electrospray ionization (ESI) mode, steroid hormones and PPCPs were detected in positive ESI mode with multiple reaction monitoring of the two most abundant product ions for each analyte. For each MRM transition, the dwell time was 40 ms and the entrance potential (EP) was 10 volts.

### Method validation

Performance was validated with respect to linearity, sensitivity, precision, recovery and freedom from matrix interferences. To evaluate isolation and LC–MS/MS analysis, pooled samples were prepared by mixing six individual killer whale faecal samples. Pooled samples were also spiked with native compounds at concentrations of 0.2, 1 and 5 ng/g. All spiked and non-spiked samples were extracted, isolated and analysed in triplicate (3 separated samples) according to the procedures described above.

### Linearity and sensitivity

Linearity was determined using seven calibration standards solutions (0.1, 0.2, 0.5, 1, 2, 5 and 10 ng/ml) prepared in 20:80 MeOH/water. To determine method limit of quantification (MLOQ), 0.006, 0.02 or 0.2 ng of each native compound was spiked into 200 μl of isolated extract, and the signal-to-noise (S/N) ratio was determined for each analyte. MLOQ was subsequently defined as the minimum analyte concentration producing an S/N ratio of at least 10:1.

### Precision and recovery

Intra-day and inter-day precision and recovery were determined using pooled unspiked faecal samples and identical samples spiked with low (5.3 ng/g), medium (26.7 ng/g) and high (133 ng/g) concentrations of each analyte. Because certified reference materials containing the 21 target compounds were not available, recoveries were determined by comparing results obtained using the spiked and unspiked samples. Intra-day experiments were conducted using nine samples (3 spiked samples for each level × 3 spiked levels). These nine samples were spiked, processed by SPE-LLE and analysed by LC–MS/MS in a single day. Inter-day experiments were also performed using nine samples (3 samples/day × 3 days). One sample at each spiked level was prepared each day and processed by SPE-LLE. All nine samples were then analysed by LC–MS/MS in a single day.

### Interference and carryover

Of the two MRM transitions selected for each analyte during LC–MS/MS, one was assigned to a quantitative (QN) product ion used for measuring analyte concentration and one to a qualitative (QL) product ion used to confirm the identity of the analyte. We used the ratio of QN and QL product ion intensities to evaluate matrix interferences. The QN:QL ratio was first measured in calibration standards and then compared with ratios obtained in faecal extracts to evaluate potential interferences from unknown compounds in these samples. To determine carryover, standard solutions containing different concentrations of each analyte (12.5 and 100 ppb) in 20:80 MeOH/water were analysed by LC–MS/MS, each followed immediately by injection of a 20:80 MeOH/water solvent blank. Analyte concentrations measured for each blank were compared with those measured in the preceding standard, a carryover of <0.5% being regarded as acceptable.

## Results and Discussion

### Method optimization

The MS/MS acquisition parameters for each compound were optimized by infusing each compound solution at 10 μl/min via the ESI source using a syringe pump. The concentration used ranged from 5 to 500 ng/ml, depending on compound of interest. The main precursor (molecular) and product (fragment) ions produced in positive and negative ionization mode during MS and MS/MS were identified. The most intense precursor-to-product ion transitions for each compound were selected for MRM and optimized for sensitivity by adjusting parameters such as the declustering potential (DP), collision energy (CE) and collision cell exit potential (CXP). The MRM transitions and MS/MS acquisition parameters are listed in Table 2 for target compounds and Table S1 for internal standards.

HPLC conditions were optimized using various mobile-phase compositions including acetonitrile/water and MeOH/water with and without the addition of formic acid. Of the solvent compositions tested, MeOH/water containing 0.1% formic acid was found to achieve best results. All 18 hormones and three PPCPs were resolved within 33 minutes by gradient elution with this solvent system, as shown in Fig. 1 for a 2 ng/ml standard mixture.

### Optimization of sample preparation

#### Extraction, concentration and isolation

The compounds of interest exhibit a wide range of polarities with octanol–water partition coefficients (log K_OW_ values) ranging from 1.08 (aldosterone) to 5.15 (sertraline). They also represent a broad diversity of acid dissociation constants (pKa values) ranging from −1.4 (DHEAs) to 19.09 (testosterone) for the most acidic functional groups within each compound and from −7.5 (DHEAs) to 9.85 (sertraline) for the most basic groups (Table S2). The effect of analyte concentration and pH on extraction efficiency was investigated by preparing, in triplicate, aqueous solutions spiked with the same amount of each analyte (0.005, 0.05 or 0.5 ppb) and adjusting the pH of each solution to a different value (2.9, 7.0 or 10.5) using acetic acid and ammonia solution. Satisfactory recoveries (85–105%) were achieved for most analytes irrespective of pH; however, best results were achieved at pH 10.5, which was subsequently used for extracting killer whale faecal samples.

Methanol and reagent alcohol were both evaluated as the initial solvent for extracting compounds of interests from whale faeces, before addition of water/pH adjustment, and produced similar results; however, reagent alcohol was selected for extraction owing to its use during field sample collection. When applying the SPE method developed using spiked water samples to alcohol extracts from faecal samples, the analyte compounds produced a much lower response during MRM analysis. Further cleanup of SPE extracts was, therefore, carried out using LLE with MTBE. In contrast to the method described by Hauser et al. (Hauser *et al.,* 2008), phase separation after LLE was achieved by centrifuging at 14 000 rpm (12 800 × g) for 10 minutes, rather than centrifuging at 1500 rpm for 5 minutes and freezing at −21°C for at least 3 hours. We found no difference between the results obtained using high-speed centrifugation and those obtained after subjecting the LLE extract to extreme cold (−80°C) for 1 hour, 2 hours and overnight, suggesting that the former provides a rapid alternative means of achieving phase separation.

#### Signal suppression

Co-elution of analyte compounds with residual matrix components may lead to suppression or apparent enhancement of the detected analyte signal. To investigate such matrix effects, seven different concentrations of native compounds (0.1–10 ng/ml) and a fixed amount of their internal standards (10 ng/ml) were prepared in pure solvent (20:80 MeOH/water), solvent containing SPE extracts, and solvent containing SPE extracts after additional cleanup by LLE with MTBE. The slopes of the calibration curves for each compound, without isotopic dilution (correction), in the solvents containing each type of extract (SPE or SPE + LLE) were compared with those obtained in pure solvent. Signal suppression was then determined by subtracting from 1 the ratio of the slope of matrix-matched standard curves to the slope of standard solution curves and multiplying this value by 100%. Results are presented in Table 3, which show that signal suppression occurred for all compounds except fluoxetine, sertraline and DHEAs for which signal enhancement was observed. However, as shown in Table 4, LLE of the SPE extracts reduced signal suppression by a factor of up to 3.8 compared with SPE alone, significantly lowering the MLOQ.

#### Isotopic dilution

After applying isotopic dilution with the available internal standards, the slopes and intercepts of the calibration curves generated for each target compound in pure solvent (20:80 MeOH/water), solvent containing SPE extracts, and solvent containing SPE extracts after LLE were determined. As can be seen from Table 5 there was relatively little variation in calibration slope between solvent media except for DHEAs (25% RSD) and 17α, 20β-dihydroxyprogesterone (13% RSD), for which isotopically labelled standards were not available. Similar slopes for the remaining compounds suggest that it may be possible to quantify them without SPE and/or LLE as long as the concentration is sufficiently high and the corresponding isotopically labelled standard is added for correction. In contrast, significant variations in the calibration intercept were observed between solvent media for all target compounds, as shown in Table 6. In most cases, the intercept for SPE + LLE was similar to or lower than that for SPE alone, a result consistent with the need for additional sample cleanup to reduce MLOQ.

### Method validation

#### Linearity and method limit of quantification

Linear calibration curves (r^2^ > 0.99) were generated from a multi-point calibration series (0.1–10 ng/ml) with a 1/X weighting fit for each target compound with Analyst software. As shown in Table 4, MLOQ ranged from 0.06 ng/g for fluoxetine to 42.52 ng/g for DHEA when using both SPE and LLE for sample cleanup before LC–MS/MS analysis. Lower sensitivity for DHEA may be due to the use of the MRM transition 289.2 > 253 for quantification rather than 271.1 > 253 (Table 2) which, although up to 3 times more intense than 289.2 > 253 in pure solvent, is subject to interference in faecal sample extracts, disqualifying it as quantification transition for DHEA.

#### Precision and recovery

Precision (% relative standard deviation) and recovery (%) were determined using samples spiked with low, medium and high concentrations of each analyte (5.3, 26.7 and 133 ng/g, respectively) as shown in Table 7. Of the 21 target compounds, all except sertraline, triclosan and estriol (which was not recovered at the lowest spiked concentration) had intra-day precisions and recoveries of 0.5–18.7% and 84.5–130.0%, respectively, and inter-day precisions and recoveries of 0.8–20.8% and 88.1–135.8%, respectively. Overall, average precision was within 10% and average recoveries between 89.3% and 129.3% for all compounds except sertraline, triclosan and estriol. Higher- or lower-than-expected recoveries may be due, in part, to lack of a corresponding labelled compound for certain analytes (e.g. 17α, 20β-dihydroxyprogesterone, for which progesterone-d_9_ was used as the internal standard). In such cases, the future availability of a more suitable internal standard may help to address this issue.

#### Interference and carryover

QN and QL precursor-to-product ion transitions were available for all analytes except triclosan to ensure quantification of the correct target compound in killer whale faecal samples. The only interference observed was for the DHEA transition 271.1 > 253, necessitating reliance on a single transition (289.2 > 253) for quantitation and confirmation of DHEA, as well as for triclosan (286.8 > 35.0). Carryover was observed for triclosan (4%) at a concentration of 100 ppb and for progesterone (4%), fluoxetine (3%) and sertraline (3%) at 12.5 ppb. However, these concentrations were much higher than those observed in unspiked samples (for which a calibration range of 0.1–10 ppb may be sufficient), although injecting MeOH between samples seemed to eliminate the carryover observed at such levels.

### Method comparison

In the absence of existing LC–MS/MS methods for hormone analysis of whale faeces, we chose to compare our method with those available for the analysis of hormones in faecal samples from other wild mammals, including New World primate species (Weltring *et al.,* 2012) and wild baboons (Gesquiere *et al.,* 2014) in terms of the number of compounds analysed and method limit of detection. As shown in Table 8, we found that the number of hormones included in our method (18) is greater than for Gesquiere et al. (6) or Weltring et al. (10). Our method limits of detection for compounds such as androstenedione, testosterone and androsterone were similar to those achieved by Gesquiere et al. (Gesquiere *et al.,* 2014), although our method was ~5 times more sensitive for estrone and 5 times less sensitive for DHEA. Our method was 6–350 times more sensitive (for 17β-E2 and cortisone, respectively) than the method previously published by Weltring et al. (Weltring *et al.,* 2012). These large differences in sensitivity may be due to differences in analytical instrumentation and/or sample matrix (i.e. killer whale vs white-faced capuchin faecal samples). However, they do suggest that our method performs relatively well in terms of quantifying multiple hormones in mammalian faecal samples. Furthermore, the inclusion of PPCPs in our method demonstrates versatility with regard to profiling different classes of compounds at the same time.

### Analysis of test samples

The optimized method was subsequently applied to the detection and quantification of hormones and PPCPs in six test samples of faecal matter obtained from killer whales under managed care. MRM chromatograms for selected analytes and the corresponding internal standards in killer whale faeces are shown in Fig. S1. The compounds 17-hydroxyprogesterone (11.4–21.8 ng/g), fluoxetine (0.6–3.6 ng/g), sertraline (1.0–11.3 ng/g) and triclosan (8.2–47.5 ng/g) were detected and measured in all samples. Cortisone (6.2 ng/g), cortisol (7.9 ng/g), androstenedione (27.5 ng/g), 17β-E2 (55.7 ng/g) and testosterone (5.1 ng/g) were also found in one sample. Analysis of wild SRKW faecal samples collected and analysed in support of the Government of Canada Whales Initiative will be reported elsewhere.

## Conclusions

A new method for simultaneous, non-invasive profiling of hormones and PPCPs in killer whales has been developed. The method is capable of quantifying 18 endogenous steroids and three PPCPs in freeze-dried killer whale faecal samples using LC–MS/MS at MLOQ ranging from 0.06 ng/g (fluoxetine) to 45.2 ng/g (DHEA). These include hormones and related metabolites that are relevant to the physiology and health of the endangered SRKW, including those associated with the HPA and HPG axes, and PPCPs to which they may be exposed to by eating Chinook salmon, their preferred prey. This method may be used to assess the health of other free-ranging killer whale populations and provides a reliable alternative to commonly used radio and enzyme immunoassays in cases where cross-reactivity and/or the number of target compounds require the specificity and flexibility of LC–MS/MS. The method can also be used to help validate new and existing immunoassays, which in some cases (for species other than *O. orca*) have been shown to produce results that differ from those obtained using LC–MS/MS (Gesquiere *et al.,* 2014; Caulfield and Padula, 2020). Physiological validations similar to those carried out during other cetacean studies (Lemos *et al.,* 2020) are recommended to further evaluate the method for assessing health and reproduction status and identifying physiological responses to disturbance in whales.

## Supplementary Material

Web_Material_coad081

## Data Availability

Data have been included with the Supplementary Material.
