# Supplementary material for: Simultaneous determination of steroid hormones and pharmaceuticals in killer whale (Orcinus orca) faecal samples by liquid chromatography tandem mass spectrometry
Source: Conserv Physiol. 2023 Nov 11;11(1):coad081. doi: 10.1093/conphys/coad081 (PMC10660373; doi:10.1093/conphys/coad081)
Supplement: Web_Material_coad081 [file web_material_coad081.pdf]

Supplementary Material

Simultaneous determination of steroid hormones and pharmaceuticals in killer whale (*Orcinus orca*) fecal samples by liquid chromatography-tandem mass spectrometry

Andrew R.S. Ross*, Xiangjun Liao and Tanya M. Brown

Fisheries and Oceans Canada, Sidney, BC, V8L 4B2, Canada

*Corresponding author: Fisheries and Oceans Canada, Sidney, BC, V8L 4B2, Canada. Tel: 1 (250) 363-6800. Email: Andrew.ross@dfo-mpo.gc.ca

Table S1: MS/MS acquisition parameters for internal standards

Compound	Precursor ion (m/z)	Product ions (m/z)	DP (volts)	CE (volts)	CXP (volts)
Estriol-d_2	291	109	121	41	20
Fluoxetine-d_5	315.3	153	81	17	22
Sertraline-d_3	309.1	275.1	31	17	14
Cortisone-d_8	369	168.4	41	35	18
Cortisol-d_2	365.1	123	161	37	52
11-Ketotestosterone-d_3	306.1	121	136	37	52
Corticosterone-d_8	355.1	125.1	70	32	10
11-Deoxycortisol-d_7	354	100	161	31	24
Androstenedione-$^{13}C_3$	290	100	61	29	18
Estrone-$^{13}C_3$	274.1	162.3	75	30	10
11-Deoxycorticosterone-d_7	338	112	91	33	48
Testosterone-d_3	292.2	97	81	31	14
17-Hydroxyprogesterone-d_8	339	100	106	33	16
DHEA-d_6	295.2	259	101	15	36
Progesterone-d_9	324.2	113	61	41	14
Androsterone-d_4	295	259	76	21	36
Triclosan-$^{13}C_{12}$	298.8	35	-150	-54	-18

DP: Declustering potential; CE: Collision energy; CXP: Collision cell exit potential

Table S2: Acid dissociation constant (pKa) and octanol-water partition coefficient (Log K_{ow}) values of measured compounds

Compound	pKa (Strongest Acidic)	pKa (Strongest Basic)	Log K_{ow}
Estriol (E3)	10.33	-3.2	2.45
Aldosterone	13.82	-2.9	1.08
Fluoextine		9.8	4.05
Sertraline		9.85	5.15
Cortisone	12.6	-3.8	1.47
Cortisol	12.58	-0.28	1.61
11-Ketotestosterone	14.79±0.60		
Corticosterone	13.86	-0.26	1.94
11-Deoxycortisol	12.59	-3.3	3.08
Androstenedione	19.03	-4.8	2.75
17ß-Estradiol (E2)	10.33	-0.88	3.57
Estrone	10.33	-5.4	3.13
11-Deoxycorticosterone	13.86	-3.3	2.88
Testosterone	19.09	-0.88	3.32
17-Hydroxyprogesterone	12.7	-3.8	2.99
DHEA (dehydroepiandrosterone)	18.2	-1.4	3.36
17α, 20β-Dihydroxyprogestetrone	14.79±0.60		
Progesterone	18.92	-4.8	3.87
Androsterone	18.3	-1.4	3.1
Triclosan	7.68	-6.7	4.76
DHEAs (Dehydroepiandrosterone sulfate)	-1.4	-7.5	3.42

pKa and log K_{ow} can be found from www.foodb.ca, except for the pKa for 11-Ketotestosterone and 17α, 20β-Dihydroxyprogestetrone which is from www.chemicalbook.com.

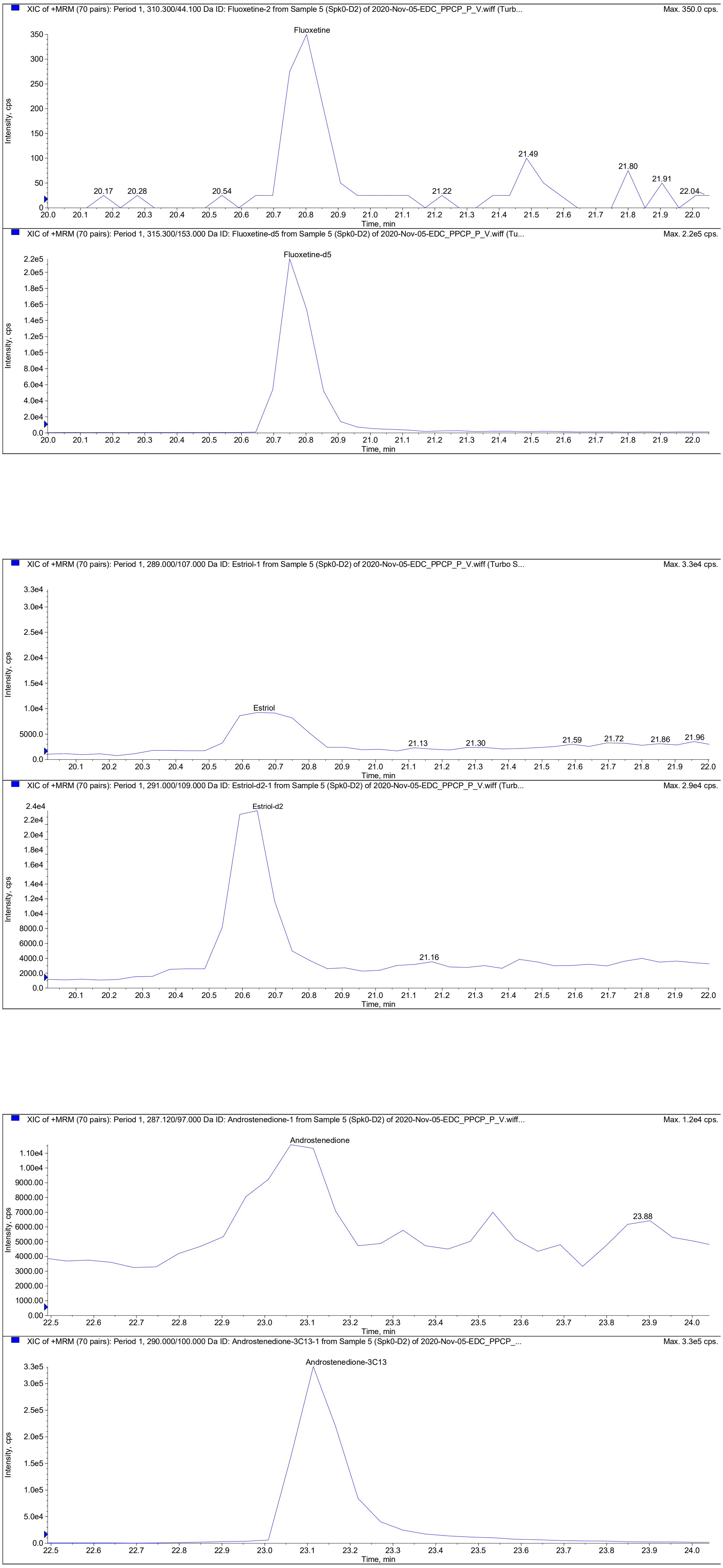

Figure S1: MRM Chromatograms for selected hormone and pharmaceutical and personal care products in killer whale feces

Chemicals and Reagents

Methanol and methyl tertiary-butyl ether (MTBE) were liquid chromatographic grade and were purchased from VWR Canada (Mississauga, ON, Canada). Reagent alcohol and formic acid were purchased from Sigma-Aldrich Canada (Oakville, ON, Canada). Double-deionized water for extraction and isolation was prepared using a Super-Q ™ water purification system (Millipore SAS, France). SPE cartridges Oasis HLB (60 mg, 3 mL) were purchased from Waters Limited (Mississauga, ON, Canada).

Native standard compounds 11-deoxycorticosterone, 11-ketotestosterone, 17β-estradiol (7β-E2), 17α, 20β-dihydroxy-4-pregnen-3-one, androstenedione, androsterone, aldosterone, cortisol (hydrocortisone), corticosterone, dehydroepiandrosterone (DHEA), dehydroepiandrosterone sulfate (sodium salt) (DHEAs), estriol and progesterone were purchased from Cayman Chemical Company (Ann Arbor, MI, USA) while cortisone, 11-deoxycortisol and 17α-hydroxyprogesterone (17-OHP) were obtained from Toronto Research Chemicals, Inc. (Toronto, ON, Canada). Native standard solutions of testosterone (100 µg/mL) in dioxane, estrone (100 µg/mL) in acetonitrile, fluoxetine:HCl (1.0 mg/mL free base) in methanol, sertraline (1 mg/mL) in methanol, and triclosan (100 µg/mL) in MTBE were supplied by ACP Chemical Inc. (Montreal, QC, Canada).

Labeled internal standards 11-deoxycorticosterone-d_7, 11-deoxycortisol-d_7, cortisone-d_8 and progesterone-d_9 were acquired from Toronto Research Chemicals, Inc. while 11-ketotestosteron-d_3 was from Cayman Chemical Company. Other deuterated internal standards including (±)-fluoxetine-d_5 HCl (phenyl-d_5), 17β-estradiol-2,4,16,16-d_4, 4-pregnen-17α-ol-3,20-dione-2,2,4,6,6,21,21,21-d_8, cortisol-1,2-d_2, 16α-hydroxy-17β-estradiol-2,4-d_2, and 4-pregnen-11β,21-diol-3,20-dione-2,2,4,6,6,17α,21,21-d_8 were purchased from C/D/N Isotopes Inc. (Pointe-Claire, QC, Canada). Labeled internal standard solutions (100 µg/mL) including testosterone (16,16,17-d_3, 98%), androsterone (5α-androstan-3α-ol-17-one) (2,2,4,4-d_4, 98%), 4-androstene-3-17-dione (2,3,4-$^{13}C_3$, 98%), dehydroepiandrosterone (DHEA) (2,2,3,4,4,6-d_6, 97%), estrone (2,3,4-$^{13}C_3$,99%), sertraline:HCl, (d_3, 98% as free base) and triclosan ($^{13}C_{12}$,

99%) were purchased from ACP Chemical Inc. Each was prepared in methanol except triclosan ($^{13}C_{12}$, 99%) which was prepared in MTBE.
